# Oliceridine and morphine differentially modulate the gut microbiota–intestinal barrier axis in a rat postoperative pain model

**DOI:** 10.3389/fphar.2026.1907666

**Published:** 2026-10-05

**Authors:** Keying Li, Sufang Jiang, Peiying Huang, Suqian Guo, Shengnan Ge, Jiaqi Li, Shizhao Wang, Lining Huang

**Affiliations:** 1 Department of Anesthesiology, The Second Hospital of Hebei Medical University, Shijiazhuang, China; 2 Hebei Key Laboratory of Neurodegenerative Disease Mechanism, Shijiazhuang, China; 3 Key Laboratory of Clinical Neurology, Ministry of Education, Hebei Medical University, Shijiazhuang, Hebei, China

**Keywords:** G protein-biased agonist, gut microbiota, intestinal barrier, oliceridine, postoperative pain, µ-opioid receptor

## Abstract

**Background:**

Oliceridine is a novel G protein-biased μ-opioid receptor agonist with potentially improved gastrointestinal tolerability compared with conventional opioids. However, its impact on postoperative intestinal homeostasis—specifically the gut microbiota and intestinal barrier—remains poorly understood.

**Methods:**

A postoperative pain model was established in male Sprague–Dawley rats using the Brennan plantar incision method. Animals were randomly assigned to four groups: non-incision control (CON), plantar incision plus normal saline, plantar incision plus oliceridine (OLI, 1 mg/kg subcutaneous bolus followed by continuous infusion at 1 mg/kg/day for 72 h), and plantar incision plus morphine (MF, 5 mg/kg subcutaneous bolus followed by continuous infusion at 5 mg/kg/day for 72 h). Analgesic efficacy was assessed by mechanical withdrawal threshold (MWT) and area under the curve (AUC). Intestinal morphology, epithelial Claudin-1 expression by immunofluorescence (n = 4 per group), gut microbiota composition (16S rRNA gene sequencing, n = 6 per group), and host ileal transcriptomic profiles (RNA-seq, n = 3 per group) were evaluated.

**Results:**

Oliceridine and morphine produced comparable analgesia, as reflected by MWT AUC (P < 0.0001 vs. NS; OLI vs. MF: P = 0.8770). Both opioids alleviated postoperative mechanical hypersensitivity. Compared with NS, opioid treatment increased the relative abundance of *Limosilactobacillus* and *Lactobacillus* and decreased *Streptococcus*. Exploratory microbiome analysis identified a potential *Lactobacillus*-related signal, but no genus-level difference between the OLI and MF groups remained significant after multiple-testing correction. Transcriptomic analysis revealed distinct ileal gene-expression profiles between the OLI and MF groups. Relative to MF, OLI was associated with higher expression of genes related to epithelial barrier integrity and brush border function and lower expression of genes involved in immune-related pathways. Histologically, both opioids attenuated intestinal injury compared with NS, with no significant difference between OLI and MF. In contrast, relative Claudin-1 fluorescence intensity was significantly higher in the OLI group than in the MF group (P = 0.036), providing protein-level support for the barrier-related transcriptomic findings. Integrated correlation analysis identified associations between *Lactobacillus*-related taxa and host genes linked to barrier function and metabolism (|r| > 0.6, P < 0.01).

**Conclusion:**

Oliceridine and morphine produced comparable analgesia. Exploratory microbiome analysis identified a potential *Lactobacillus*-related signal, but no genus-level differences remained significant after multiple-testing correction. Oliceridine was associated with a distinct ileal transcriptomic profile and higher Claudin-1 fluorescence intensity than morphine, although histological injury did not differ between the two groups. The functional significance of these molecular differences requires further investigation.

## 1 Introduction

Opioids remain the mainstay of perioperative and postoperative pain management. However, their use is limited by adverse effects including respiratory depression, nausea, vomiting, and gastrointestinal dysfunction (Feng et al., 2024). Among these, opioid-induced gastrointestinal adverse effects notably delay postoperative recovery and represent a major barrier to long-term opioid therapy (Kistemaker et al., 2024).

Numerous studies have confirmed that opioids broadly disrupt gastrointestinal physiology (Akbarali and Dewey, 2019), manifesting as inhibited motility, impaired intestinal mucosal barrier integrity (Jalodia et al., 2022; Ma et al., 2025), and disturbed gut microbiota homeostasis (Banerjee et al., 2016; Wang et al., 2018). Opioid-induced dysbiosis is characterized by reduced abundance of potentially beneficial bacteria such as *Lactobacillus* and *Bifidobacterium*, along with overgrowth of opportunistic pathogens like *Enterococcus*. Multi-omics analyses have further elucidated these microbial and molecular alterations (Zhang et al., 2019; Kolli et al., 2023).

Receptor agonists can be designed to preferentially activate specific intracellular signaling pathways—a concept known as biased agonism. The μ-opioid receptor (MOR) mediates opioid analgesia through multiple intracellular signaling pathways, including G protein- and β-arrestin-associated signaling. Excessive β-arrestin pathway activation has been proposed to contribute to several opioid-related adverse effects, including respiratory depression, tolerance, and constipation (Madariaga-Mazón et al., 2017). This has prompted the development of MOR-biased agonists that aim to enhance analgesia while minimizing side effects (Siuda et al., 2017).

Oliceridine is a novel G protein-biased MOR agonist that preferentially activates G protein signaling with attenuated β-arrestin recruitment (Yudin and Rohacs, 2019; Markham, 2020). Preclinical and clinical studies suggest that oliceridine may have a more favorable gastrointestinal tolerability profile than conventional opioids such as morphine, as evidenced by reduced inhibition of gastrointestinal motility (DeWire et al., 2013; Altarifi et al., 2017) and a lower incidence of postoperative gastrointestinal adverse events (Singla et al., 2019; Daksla et al., 2023; Biskupiak et al., 2024; Meng et al., 2025; Zeng et al., 2026). However, the effects of oliceridine on gut microbiota composition and intestinal barrier-related features remain largely unexplored, and direct comparisons with classical opioids under clinically relevant conditions are lacking.

To address this gap, we employed a rat plantar incision model with continuous opioid administration for 72 h to simulate sustained postoperative analgesia. At doses that produced comparable analgesia, we compared the effects of oliceridine and morphine on postoperative pain behavior, gut microbial composition, intestinal histopathology, and host ileal transcriptional responses. By integrating behavioral, microbiome, and transcriptomic analyses, this study aimed to characterize potential differences in intestinal responses associated with a G protein-biased MOR agonist and conventional opioids during postoperative analgesia.

## 2 Methods

### 2.1 Experimental animals

All animal procedures were approved by the Animal Care and Welfare Ethics Committee of the Second Hospital of Hebei Medical University (Approval No.2025-AE422) and conducted in accordance with the National Institutes of Health Guide for the Care and Use of Laboratory Animals. Male Sprague–Dawley rats (6–8 weeks old, 200–240 g) were obtained from Beijing HFK Bioscience Co., Ltd. (Beijing, China). Animals were housed under specific pathogen-free conditions with a 12-h light/dark cycle, controlled temperature (23 °C ± 2 °C), and relative humidity (55% ± 5%). Food and water were provided *ad libitum*, with three to four rats per cage. All experimental procedures, group allocations, and outcome assessments were performed by investigators blinded to treatment conditions. Animals were randomized using a computer-generated random sequence. Cage allocation was balanced across groups. Investigators responsible for behavioral testing, histological scoring, sequencing analysis, and statistical analysis were blinded to group allocation. No animals were excluded unless predefined humane endpoints were met.

### 2.2 Experimental design and grouping

Animals were randomly assigned to experimental groups (n = 10 per group) using a computer-generated randomization sequence, and cage allocation was balanced across groups. Investigators responsible for behavioral testing, histological scoring, and immunofluorescence image quantification were blinded to group allocation. No animals were excluded unless predefined humane endpoints were met. The sample sizes were selected based on previous studies using the Brennan plantar incision model and microbiome endpoints. Behavioral assessments were performed in all 10 animals per group. Subsets of animals were used for 16S rRNA gene sequencing (n = 6 per group), RNA sequencing (n = 3 per group), histological analysis (n = 3 per group), and Claudin-1 immunofluorescence analysis (n = 4 per group). Animals used for RNA sequencing were included among those used for 16S rRNA gene sequencing, allowing paired microbiome–transcriptome analysis.

The experimental groups were defined as follows:

1. Non-incision control group (CON group): Rats received sevoflurane anesthesia without plantar incision, followed by an initial subcutaneous injection of an equivalent volume of normal saline and continuous subcutaneous infusion of normal saline using an osmotic minipump.
2. Plantar incision plus normal saline group (NS group): Rats underwent plantar incision under sevoflurane anesthesia, followed by an initial subcutaneous injection of an equivalent volume of normal saline and continuous subcutaneous infusion of normal saline using an osmotic minipump.
3. Plantar incision plus oliceridine group (OLI group): Rats underwent plantar incision under sevoflurane anesthesia, followed by an initial subcutaneous bolus of oliceridine and continuous subcutaneous infusion of oliceridine using an osmotic minipump.
4. Plantar incision plus morphine group (MF group): Rats underwent plantar incision under sevoflurane anesthesia, followed by an initial subcutaneous bolus of morphine and continuous subcutaneous infusion of morphine using an osmotic minipump.

### 2.3 Rat model of postoperative incisional pain

#### 2.3.1 Acclimatization and basal level measurement

Rats were allowed 7 days to become accustomed to the colony room, gently handled daily for 5 min, and left in the test room for 2 h. All experiments were performed by an investigator blinded to the drugs administered. Basal value for each test was the mean of three consecutive measurements performed at day −2, −1 and 0, before surgery, with 3-min intervals (Figure 1a).

**FIGURE 1:**
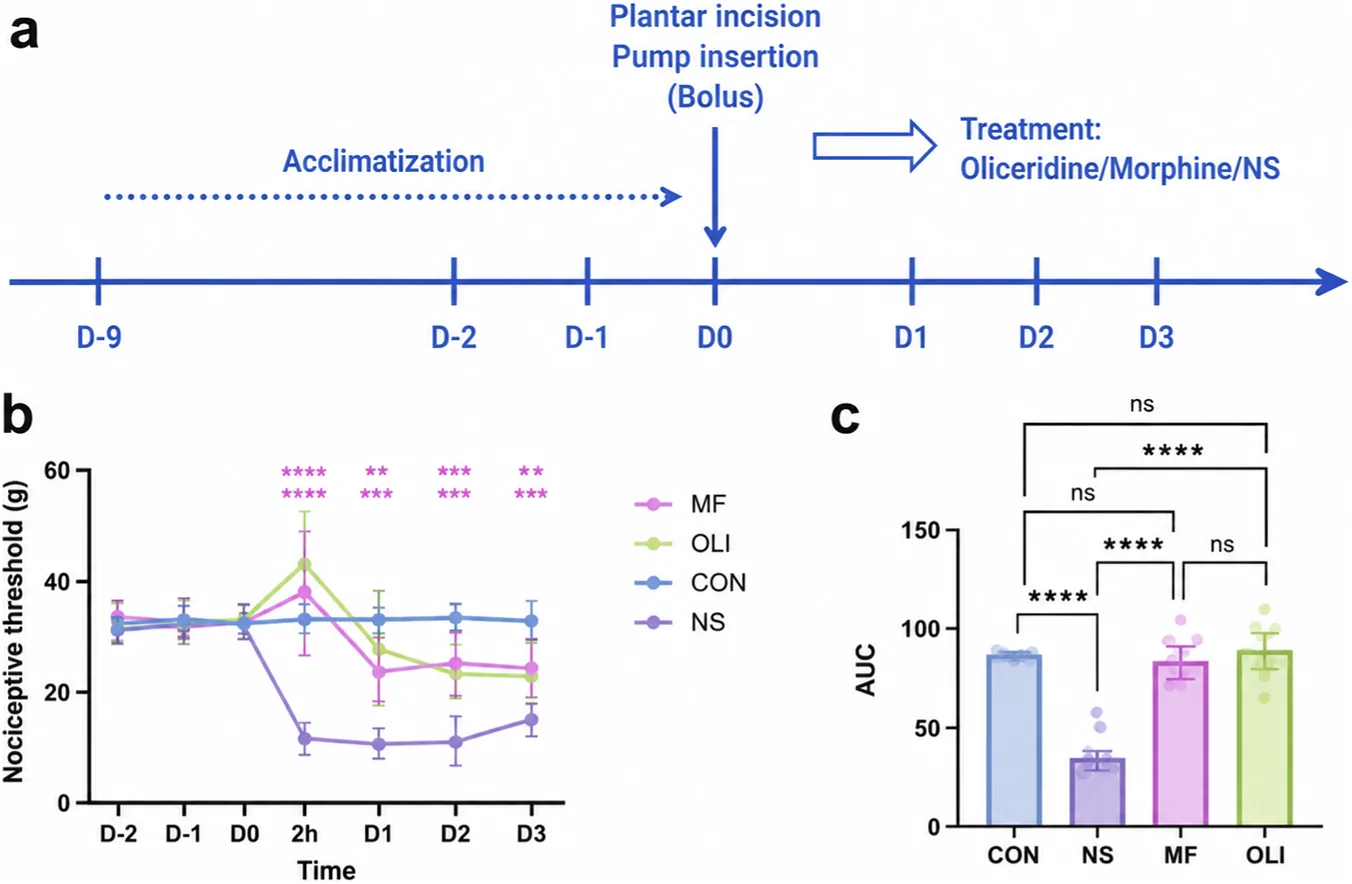
Experimental protocol and effects of opioids on mechanical withdrawal threshold (MWT) in rats after plantar incision. **(a)** Experimental protocol. Immediately after RWD pump implantation and plantar incision, rats received an initial subcutaneous bolus of the assigned treatment followed by continuous infusion via osmotic minipumps for 72 h, whereas vehicle-treated groups received saline solution. Mechanical paw withdrawal thresholds were measured using a rat mechanical nociceptive tester before (D−2, D−1, and D0) and after (2 h, D1, D2, and D3) plantar incision by the same blinded investigator. **(b)** Time course of MWT on the ipsilateral hind paw. Paw incision at D0 induced marked mechanical hyperalgesia in the NS group, which was significantly attenuated by OLI and MF at 2 h and throughout the postoperative period (P < 0.0001 vs. NS). **(c)** Area under the curve (AUC) of MWT from 2 h to postoperative day 3 (D3). Both OLI and MF significantly increased AUC compared with NS (P < 0.0001), with no significant difference between OLI and MF (P = 0.8770). Data are presented as mean ± SD (n = 10 per group). MWT data were analyzed using two-way repeated-measures ANOVA followed by Tukey’s multiple-comparisons test. AUC data were analyzed using one-way ANOVA followed by Tukey’s multiple-comparisons test.

#### 2.3.2 Surgical procedure

All rats underwent osmotic minipump implantation under general anesthesia induced and maintained with sevoflurane (3% in oxygen via a nose cone) as previously described (Hazam et al., 2024). Following minipump implantation, rats in the NS, OLI, and MF groups underwent plantar incision, whereas rats in the CON group received anesthesia and minipump implantation without plantar incision. Osmotic minipumps (RWD Life Science, Shenzhen, China) were filled with vehicle (normal saline), oliceridine, or morphine solution. Prior to implantation, pumps were primed in sterile saline at 37 °C for 16–18 h to ensure stable drug delivery. Minipumps were implanted subcutaneously in the left dorsal flank. A small subcutaneous pocket was created to accommodate the pump, which was secured in place before the incision was closed with silk sutures. Immediately following implantation, a plantar incision was performed as described by Brennan et al. (1996). Briefly, the plantar surface of the left hind paw was prepared aseptically for surgery. The incisional pain model was created by making a longitudinal incision through the skin and fascia of the plantar aspect of the left hind paw, starting 0.5 cm from the proximal edge of the heel and extending 1 cm. The plantaris muscle was isolated, elevated, and then incised longitudinally. The skin was closed, and the wound was covered with an antibiotic ointment. Then, the rats were returned to the housing cages and were allowed to recover from general anesthesia. They were monitored for any adverse reactions to the surgery. Pain behaviors were tested as early as 2 h after incision.

#### 2.3.3 Drug administration

To ensure rapid initial exposure followed by sustained drug delivery, opioids were administered via a combined bolus–continuous infusion regimen (Hazam et al., 2024; Liu et al., 2024; Wang et al., 2024). Immediately after completion of the assigned procedures, rats in the OLI and MF groups received a single subcutaneous bolus of oliceridine (1 mg/kg) or morphine (5 mg/kg), respectively. Rats in the CON and NS groups received an equivalent volume of sterile saline by subcutaneous injection. The opioid doses were selected based on previously reported analgesic doses of oliceridine and morphine (Zahn et al., 1997; Altarifi et al., 2017; Liang et al., 2019; Blakeley-Ruiz et al., 2022). For sustained postoperative analgesia, RWD 2 mL1W osmotic minipumps (RWD Life Science, China) were implanted subcutaneously to deliver morphine (5 mg/kg/day, conventional analgesic dose), oliceridine (1 mg/kg/day), or normal saline at 10 μL/h for 72 h. All drug solutions were prepared in sterile normal saline under aseptic conditions. Plasma drug concentrations were not measured; however, the behavioral assessments confirmed that both opioid regimens produced significant analgesic effects.

#### 2.3.4 Behavioral measurements

Postoperative mechanical hypersensitivity was evaluated using stimulus-evoked behavioral assays in accordance with established protocols for the plantar incision model. Behavioral testing was performed at predefined time points by investigators blinded to treatment conditions.

Mechanical Nociceptive Testing. Mechanical sensitivity was assessed using the rat mechanical nociceptive tester (Suzhou Snsek Medical Limited). Briefly, rats were placed on an elevated perforated metal platform and individually enclosed in transparent Plexiglas chambers, allowing access to the plantar surface of the hind paw. The probe tip was applied perpendicularly to the plantar surface of the left hind paw, approximately 3–4 mm medial to the surgical incision site. Force was gradually increased until paw withdrawal was elicited. The paw withdrawal threshold was defined as the force (g) required to evoke withdrawal of the paw in response to mechanical stimulation. At each time point, three consecutive measurements were obtained with a 3-min interval between trials. A cutoff force of 50 g was applied to prevent tissue injury. The mean of the three measurements was used for subsequent statistical analysis.

### 2.4 Sample collection

At 72 h postoperatively, rats were euthanized by CO_2_ inhalation in a dedicated chamber at a flow rate of 30%–70% of chamber volume per minute, followed by cervical dislocation to confirm death. Luminal contents from the terminal ileum were gently flushed with sterile saline, collected under aseptic conditions, and immediately flash-frozen in liquid nitrogen for subsequent 16S rRNA gene sequencing. Ileal tissue samples were harvested and either snap-frozen for RNA extraction and subsequent transcriptome sequencing or fixed in 10% neutral-buffered formalin for histological analysis. All euthanasia procedures were performed in accordance with the AVMA Guidelines for the Euthanasia of Animals (2020 edition).

### 2.5 Histological analysis

To assess the effects of morphine and oliceridine on intestinal morphology, distal terminal ileal tissues were collected immediately after euthanasia. Samples were fixed in 10% neutral-buffered formalin, embedded in paraffin, and sectioned at a thickness of 8 μm. Sections were mounted on glass slides and stained with hematoxylin and eosin (H&E) for histopathological evaluation. Histological scoring was performed using ileal sections from three biological replicates per group. All histological examinations and scoring procedures were performed in a blinded manner. Histological injury was assessed using a composite scoring system based on cumulative scores assigned to four histological features (Jalodia et al., 2022), including villus architecture, epithelial damage, inflammatory cell infiltration, and epithelial shedding into the intestinal lumen, as described in Supplementary Figure S1. The total histological score ranged from 0 (normal morphology) to 13 (severe tissue injury and inflammatory cell infiltration), with higher scores indicating more severe intestinal histopathological alterations.

### 2.6 Immunofluorescence

Distal ileal tissues from four animals per group were fixed in 10% neutral-buffered formalin, embedded in paraffin, and sectioned at 5 μm. After deparaffinization, rehydration, and antigen retrieval with citrate buffer, sections were blocked and incubated overnight at 4 °C with a mouse monoclonal anti-Claudin-1 antibody (clone 2H10D10; Invitrogen, Carlsbad, CA, United States; Cat. No. 37-4900; 1:500), followed by incubation with a fluorescently labeled anti-mouse IgG secondary antibody. Nuclei were counterstained with DAPI, and fluorescent images were acquired using a confocal laser-scanning microscope (Olympus FV1200) with a 20× objective under identical imaging settings. Claudin-1 fluorescence intensity within the villus epithelial region was quantified using ImageJ software (version 1.42q; National Institutes of Health, Bethesda, MD, United States). Multiple fields were averaged to obtain one value per animal, and the values were normalized to the mean of the CON group, which was set to 1.

### 2.7 16S rRNA sequencing

Ileal luminal contents were collected for gut microbiota analysis. Total microbial DNA was extracted using the OMEGA Soil DNA Kit (M5635-02; Omega Bio-Tek, Norcross, GA, United States). The V3-V4 region of the bacterial 16S rRNA gene was amplified using the 338F/806R primer pair and sequenced on the Illumina NovaSeq 6000 platform using 2 × 250-bp paired-end reads. Raw sequences were processed using QIIME2 version 2022.11. Following demultiplexing and primer removal, reads were quality-filtered, denoised, merged, and screened for chimeras using DADA2 to generate amplicon sequence variants (ASVs). Taxonomic classification was performed against the Genome Taxonomy Database (GTDB).

Alpha- and beta-diversity analyses were conducted using QIIME2 and R. Beta-diversity was evaluated using Bray–Curtis distances and visualized by non-metric multidimensional scaling (NMDS). Within-group Bray–Curtis distances were compared using the Kruskal–Wallis test followed by Dunn’s *post hoc* test. Differentially abundant taxa were explored using linear discriminant analysis effect size (LEfSe), with an unadjusted P value < 0.05 and an LDA score threshold of 2.0. To evaluate genus-level differences between the OLI and MF groups while accounting for multiple testing, the relative abundance of each of the 450 genera was compared using the Kruskal–Wallis test, and the resulting P values were adjusted using the Benjamini–Hochberg procedure. An FDR-adjusted P value < 0.05 was considered statistically significant. Additional procedures are described in the Supplementary Methods.

### 2.8 RNA sequencing

Distal ileum tissues from the four experimental groups (n = 3 per group) were collected for transcriptomic analysis. Whole distal ileal tissue was used without separation of the intestinal wall layers or enrichment of specific cell populations. Total RNA was extracted using TRIzol reagent, and RNA quality was assessed before library construction. Sequencing libraries were prepared following mRNA enrichment, RNA fragmentation, cDNA synthesis, adaptor ligation, size selection, and PCR amplification and were subsequently sequenced on the Illumina NovaSeq 6000 platform. Raw reads were quality-filtered using fastp version 0.22.0, and clean reads were aligned to the rat reference genome using STAR. Gene-level raw read counts were generated using HTSeq version 0.9.1. Differential expression analysis was performed using DESeq2 version 1.38.3 based on raw count data, with the primary comparison focusing on the OLI and MF groups. Genes with an absolute log2 fold change > 1 and a Benjamini-Hochberg-adjusted P value < 0.05 were considered differentially expressed. FPKM values were used only for gene-expression visualization and were not used for differential expression testing. Functional enrichment analysis was performed using topGO version 2.50.0. GO terms with a Benjamini-Hochberg-adjusted P value < 0.05 were considered significantly enriched. Additional procedures are described in the Supplementary Methods.

### 2.9 Statistical analysis

Statistical analyses were performed using GraphPad Prism 10.0 (GraphPad Software, San Diego, CA, United States). All data are presented as mean ± standard deviation (SD), unless otherwise specified. Normality was assessed using the Shapiro–Wilk test. Behavioral data collected at multiple time points were analyzed using two-way repeated-measures ANOVA, with treatment and time as factors, followed by Tukey’s multiple comparisons test. Single-time-point measurements, including the area under the curve (AUC) of the mechanical withdrawal threshold, histological injury scores, and relative Claudin-1 fluorescence intensity, were analyzed using one-way ANOVA followed by Tukey’s multiple comparisons test. A two-sided P value < 0.05 was considered statistically significant. Detailed statistical procedures for the microbiome and transcriptomic analyses are described in the Supplementary Methods.

## 3 Results

### 3.1 Oliceridine exerted an analgesic effect comparable to that of morphine in the rat model of postoperative acute pain

To investigate the analgesic effects of oliceridine and morphine in managing postoperative pain, we chose to assess them using the Brennan plantar incision model (Brennan et al., 1996). Based on the fact that hyperalgesia generated by plantar incision in the rat’s hind paw peaks at 2 h post-surgery and lasts 2–4 days before spontaneous remission, we therefore opted to perform measurements of the behavioral response at 2 h, day 1, day 2 and day 3 post-incision. In that respect, opioids were administered by continuous infusion using RWD pump, preceded by an additional bolus injection of these drugs, and were compared with continuous infusion of vehicle (saline solution). The antihyperalgesic effect of opioids was first evaluated using a rat mechanical nociceptive tester, which is commonly used to assess mechanical allodynia-like behavior in rodents in response to mechanical stimulation. Mechanical withdrawal threshold (MWT) was determined as the force required to elicit paw withdrawal. Mechanical withdrawal threshold (MWT) was assessed on the ipsilateral hind paw using a rat mechanical nociceptive tester with a maximum cutoff force of 50 g to avoid tissue injury. This assay was performed in four groups of rats, and dynamic changes in MWT were recorded over time. As shown in Figure 1b, baseline MWT values measured on day −2 (D−2), day −1 (D−1), and pre-incision on day 0 (D0) did not differ significantly among groups. Paw incision at D0 induced marked mechanical hyperalgesia, as evidenced by a rapid reduction in MWT at 2 h after surgery. In the vehicle group, MWT decreased from 32.26 g at D0 to 12.17 g at 2 h post-incision, indicating successful establishment of postoperative pain. In contrast, administration of either opioid significantly reduced mechanical hyperalgesia throughout the postoperative observation period compared with the NS group (P < 0.0001), as indicated by elevated MWT values. Direct comparison between the oliceridine and morphine groups revealed no significant differences in MWT at any postoperative time point (2 h: P = 0.7062; D1: P = 0.7444; D2: P = 0.8954; D3: P = 0.9245), indicating comparable analgesic efficacy in this model. To evaluate overall analgesic performance across the postoperative period, the area under the curve (AUC) of MWT from 2 h to D3 was calculated. Both opioid groups showed significantly greater AUC values than the NS group (OLI vs. NS: P < 0.0001; MF vs. NS: P < 0.0001), whereas no significant difference was detected between the oliceridine and morphine groups (P = 0.8770). The results showed that both opioids produced comparable analgesic effects in this postoperative pain model, with no significant difference in MWT between the oliceridine- and morphine-treated groups.

### 3.2 Postoperative pain and opioid analgesia were associated with distinct small intestinal microbial profiles

To investigate the effects of postoperative pain and opioid analgesia on small intestinal microbial composition, 16S rRNA gene sequencing was performed in the CON, NS, MF, and OLI groups. At the phylum level, exploratory LEfSe analysis indicated a higher relative abundance of *Firmicutes* in both opioid-treated groups than in the NS group. At the genus level, the OLI and MF groups showed higher relative abundances of *Limosilactobacillus* and *Lactobacillus* and a lower relative abundance of *Streptococcus* than the NS group (Figures 2a,d,e). NMDS ordination showed visual separation of microbial community profiles among the four groups (Figure 2b). The distributions of within-group microbial community distances differed significantly among the four groups (Figure 2c, Kruskal–Wallis test, P = 0.00021). Significant pairwise differences were further identified using Dunn’s *post hoc* test, as indicated in Figure 2c. In contrast, alpha-diversity indices did not differ significantly among the groups, indicating no detectable differences in microbial richness or evenness (Supplementary Figure S2).

**FIGURE 2:**
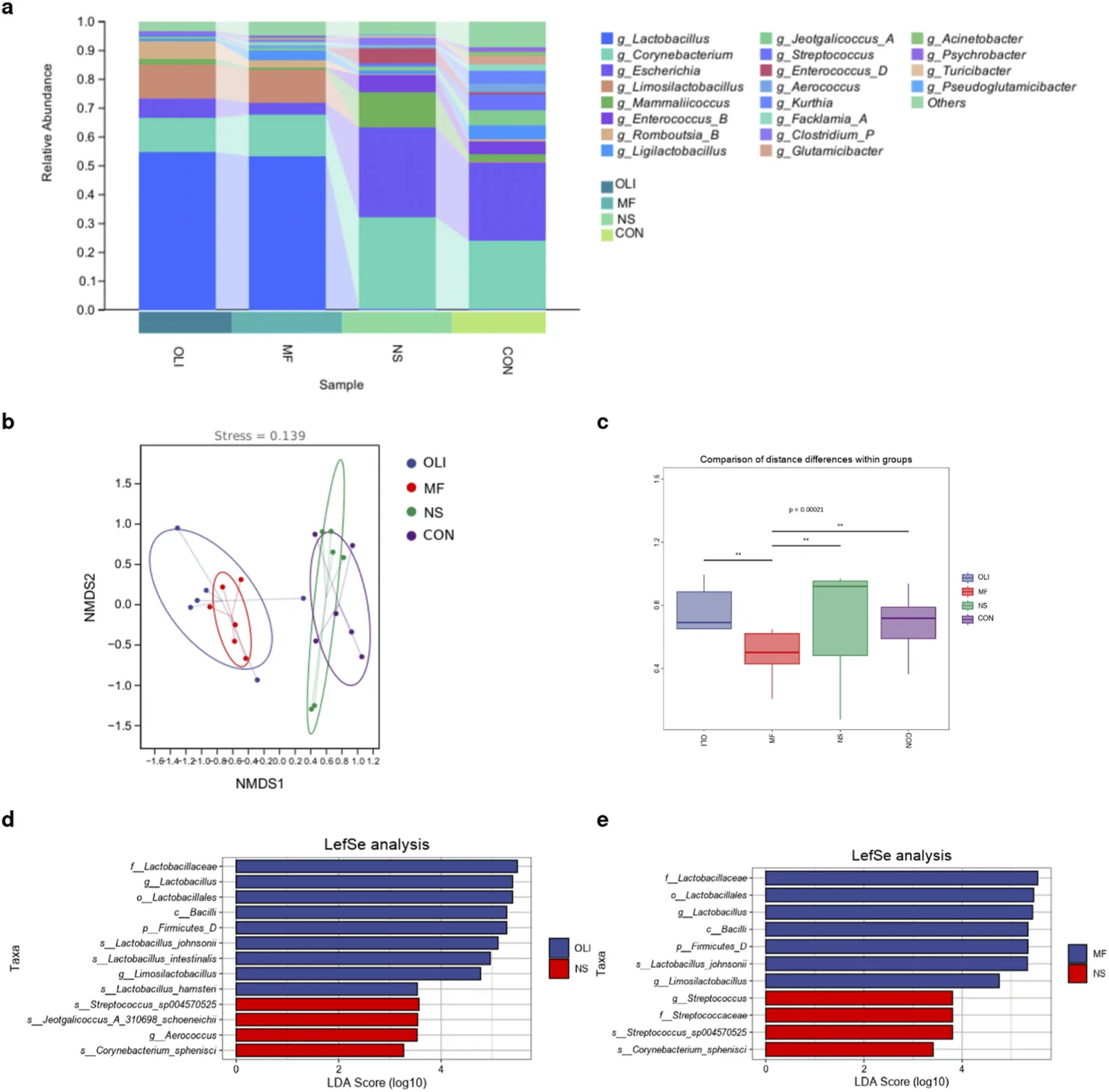
Gut microbial profiles following plantar incision and opioid treatment. **(a)** Genus-level microbial composition. **(b)** NMDS ordination based on Bray–Curtis distances. **(c)** Comparison of distance differences within groups. The overall difference was significant (Kruskal–Wallis P = 0.00021), followed by Dunn’s *post hoc* test. Significant pairwise comparisons and the corresponding adjusted P values are indicated in the figure. **(d,e)** LEfSe analysis of OLI versus NS **(d)** and MF versus NS **(e)**, using an unadjusted P < 0.05 and an LDA score threshold of 2.0. n = 6 per group. CON, control; NS, plantar incision plus normal saline; OLI, oliceridine; MF, morphine.

Exploratory LEfSe analysis was performed using an unadjusted P < 0.05 and an LDA score threshold of 2.0. The taxonomic signatures identified in the OLI-versus-NS and MF-versus-NS comparisons are shown in Figures 2d,e. In the exploratory OLI-versus-MF comparison, a *Lactobacillus*-related taxon annotated as *Lactobacillus hamsteri* was identified as a potential discriminator (Supplementary Figure S3).

To account for multiple testing, all 450 genera were additionally compared between the OLI and MF groups using the Kruskal–Wallis test followed by Benjamini–Hochberg correction. Two genera showed nominal differences at an unadjusted P < 0.05, but none remained significant after FDR correction. The relative abundance of *Lactobacillus* did not differ significantly between the OLI and MF groups (Kruskal–Wallis P = 0.5218, FDR-adjusted P = 1.0000). Therefore, the species-level LEfSe signal should be considered exploratory rather than confirmatory. Its taxonomic assignment should also be interpreted cautiously because of the limited species-level resolution of short-read V3-V4 16S rRNA gene sequencing. Collectively, postoperative pain and opioid analgesia were associated with distinct small intestinal microbial profiles. However, no genus-level difference between the OLI and MF groups remained significant after multiple-testing correction.

### 3.3 Oliceridine was associated with a distinct ileal transcriptomic profile and greater preservation of epithelial Claudin-1 expression

To further investigate the differential effects of oliceridine and morphine on the intestinal response to postoperative pain, transcriptomic analysis of ileal tissues was performed using RNA sequencing. Principal component analysis (PCA) demonstrated clear separation between the MF and OLI groups, indicating distinct transcriptomic profiles in the ileum following treatment with the two opioids (Figure 3a). Differential expression analysis identified a set of differentially expressed genes (DEGs) between the MF and OLI groups after correction for multiple testing (Figures 3b,c). Gene Ontology (GO) enrichment analysis further demonstrated distinct functional patterns between genes downregulated and upregulated in the MF group relative to the OLI group. Genes downregulated in the MF group were significantly enriched in GO terms associated with the brush border and brush border membrane, suggesting potential alterations in epithelial barrier-related molecular features (Figure 3e). In addition, these genes were enriched in multiple metabolic processes, including steroid, lipid, and alcohol biosynthetic processes (Figure 3e). Consistent with the GO enrichment results, selected brush border-associated genes showed generally higher expression in the OLI group than in the MF group (Figure 3d). In contrast, genes upregulated in the MF group were significantly enriched in immune-related GO terms, including antigen processing and presentation and MHC-related processes (Figure 3f). All reported GO enrichment results were significant after Benjamini–Hochberg correction for multiple testing.

**FIGURE 3:**
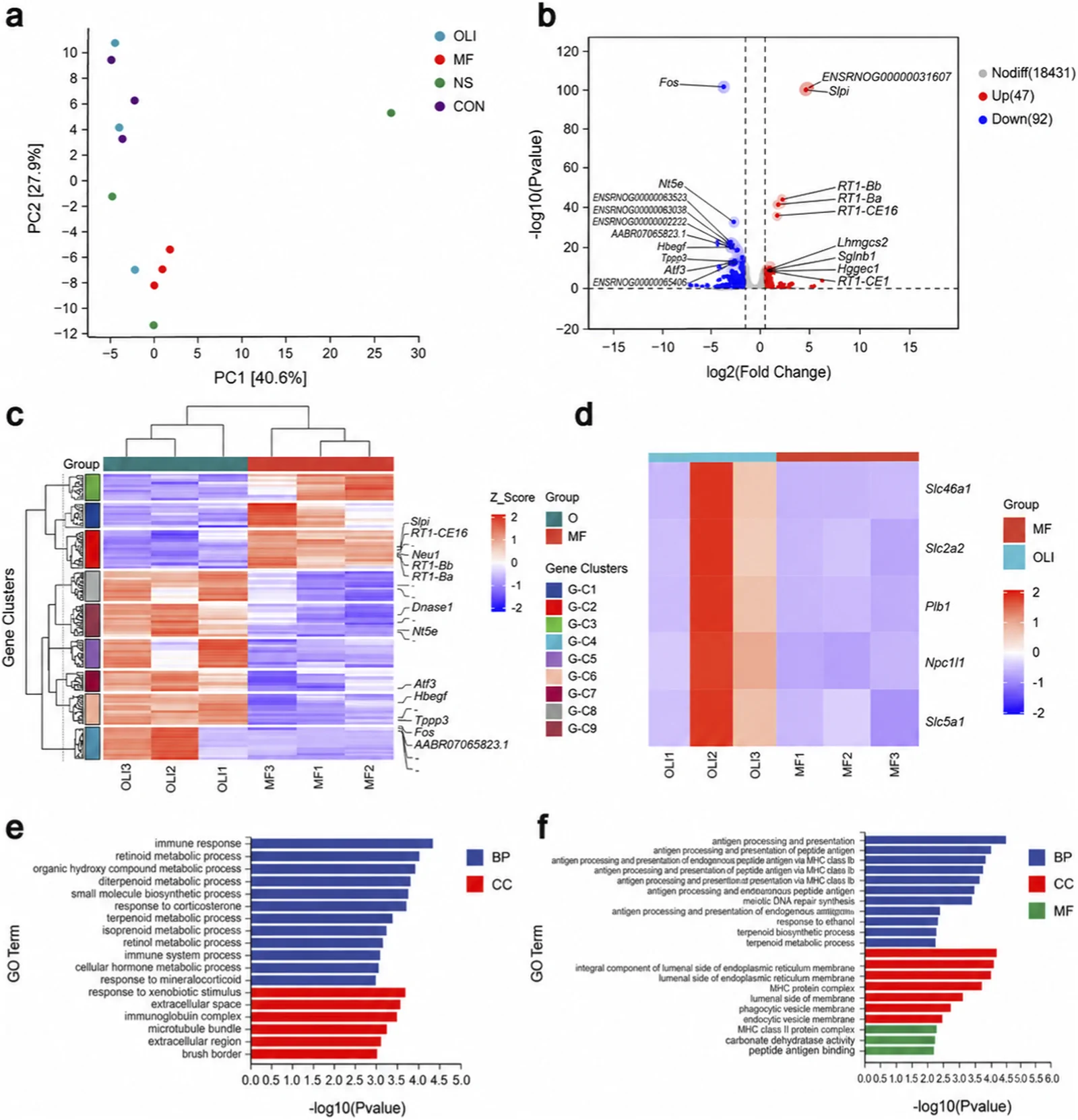
Oliceridine was associated with a distinct ileal transcriptomic profile involving epithelial barrier-related and metabolic pathways. **(a)** Principal component analysis (PCA) of ileal transcriptomic profiles. **(b)** Volcano plot of differentially expressed genes (DEGs) between the oliceridine (OLI) and morphine (MF) groups. **(c)** Heatmap of DEGs between the OLI and MF groups. **(d)** Heatmap showing the expression patterns of selected brush border-associated genes. **(e,f)** GO enrichment analysis of genes downregulated **(e)** and upregulated **(f)** in the MF group relative to the OLI group. DEGs were defined as |log2 fold change| > 1 and Benjamini-Hochberg-adjusted *P* < 0.05. GO enrichment results were also corrected using the Benjamini–Hochberg method, with adjusted *P* < 0.05 considered significant. n = 3 biological replicates per group.

To evaluate whether these transcriptomic alterations were associated with detectable structural changes, distal ileal sections were evaluated histologically. Representative H&E-stained sections demonstrated histopathological changes in the NS group, characterized by altered villus architecture, epithelial damage, inflammatory cell infiltration, and epithelial shedding into the lumen (Figure 4a). Both opioid-treated groups exhibited reduced histopathological abnormalities compared with the vehicle-treated pain group. Consistent with these observations, intestinal injury scores were significantly higher in the NS group than in the CON group (P < 0.01), whereas both opioid-treated groups showed significantly lower injury scores than the NS group and scores that were not significantly different from those of the CON group (Figure 4b). No significant difference in histological injury scores was observed between the MF and OLI groups. To further evaluate epithelial tight junctions at the protein level, Claudin-1 expression in the distal ileum was examined by immunofluorescence staining (Figure 4c). Claudin-1 fluorescence was predominantly distributed along the villus epithelium. Quantitative analysis showed that relative Claudin-1 fluorescence intensity was lowest in the NS group and highest in the CON group. Both opioid-treated groups exhibited significantly greater Claudin-1 fluorescence intensity than the NS group, whereas the OLI group showed significantly greater fluorescence intensity than the MF group (P = 0.036; Figure 4d). These findings suggest that oliceridine was associated with greater preservation of epithelial Claudin-1 immunoreactivity than morphine. Collectively, these findings indicate that, compared with morphine, oliceridine was associated with differential regulation of epithelial barrier-related, immune-related, and metabolic pathways, together with greater preservation of epithelial Claudin-1 expression, despite the absence of a detectable difference in conventional histological injury between the two opioid-treated groups.

**FIGURE 4:**
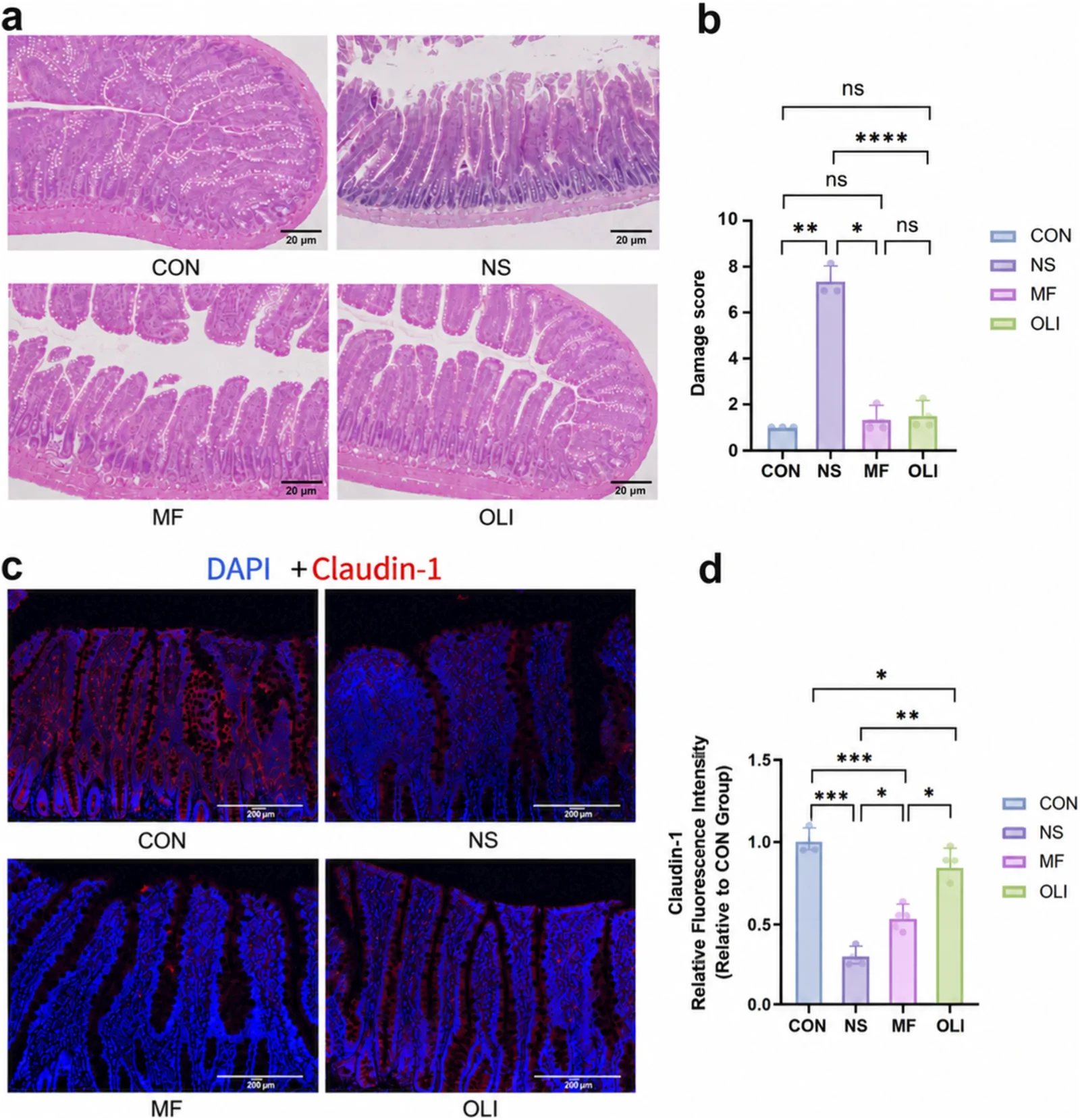
Histopathological assessment and Claudin-1 immunofluorescence analysis of the distal ileum following plantar incision and opioid treatment. **(a)** Representative H&E-stained distal ileal sections from each experimental group (100× magnification; scale bar = 20 μm). **(b)** Quantification of histological injury scores using a composite scoring system (n = 3 biological replicates per group). **(c)** Representative immunofluorescence images of Claudin-1 expression in the distal ileum. Claudin-1 is shown in red, and nuclei were counterstained with DAPI in blue. Scale bar = 200 μm. **(d)** Quantification of relative Claudin-1 fluorescence intensity normalized to the mean value of the CON group (n = 4 biological replicates per group). Data are presented as mean ± SD. Statistical analysis was performed using one-way ANOVA followed by Tukey’s multiple comparisons test. *P < 0.05, **P < 0.01, ***P < 0.001, and ****P < 0.0001 for the comparisons indicated by the connecting lines.

### 3.4 Integrated microbiome–transcriptome analysis identified associations between microbial taxa and host intestinal gene expression

To explore the associations between gut microbiota alterations and intestinal transcriptomic profiles following treatment with different opioid analgesics, Spearman correlation analysis was performed between the relative abundances of microbial taxa identified by LEfSe and the normalized expression levels of differentially expressed genes (DEGs). Correlation pairs with |r| > 0.6 and a nominal P < 0.01 were retained to construct an exploratory microbiome–transcriptome association network, which was visualized using Gephi. To improve readability, only nodes with a degree ≥ 4 in the complete correlation network were displayed (Figure 5). Pink and blue nodes represent microbial taxa and DEGs, respectively, whereas red and blue edges indicate positive and negative correlations, respectively. Node size is proportional to degree, representing the number of associations connected to each node.

**FIGURE 5:**
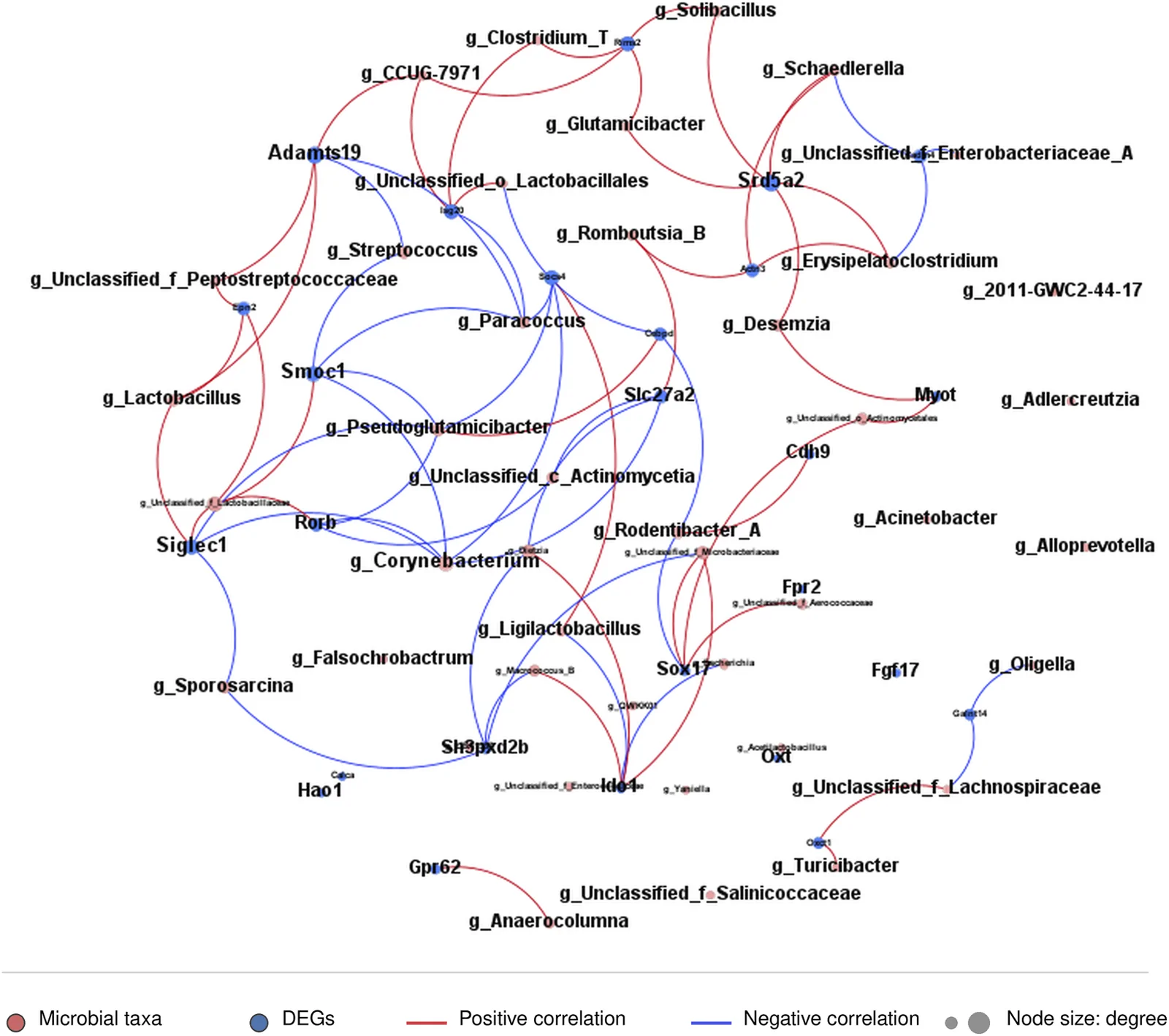
Core correlation network between gut microbial taxa and intestinal differentially expressed genes. Spearman correlations with |r| > 0.6 and a nominal P < 0.01 are shown. For visualization, nodes with a degree ≥ 4 in the complete correlation network were retained. Pink and blue nodes represent microbial taxa and differentially expressed genes (DEGs), respectively. Red and blue edges indicate positive and negative correlations, respectively, and node size is proportional to degree. The displayed correlations are exploratory and were not adjusted for multiple testing. The analysis included 12 paired microbiome–transcriptome samples from the four groups (n = 3 per group).

The resulting network revealed multiple positive and negative associations between microbial taxa and intestinal DEGs. Notably, *Lactobacillus*-related taxa were positively correlated with several intestinal DEGs, highlighting exploratory associations between *Lactobacillus*-related taxa and host transcriptional patterns. These findings should be interpreted as exploratory associations and do not establish causal relationships between microbial alterations and intestinal gene expression.

## 4 Discussion

Previous studies have confirmed that analgesic doses of morphine significantly alter the gut microbiome structure and cause adverse effects on the ileum (Rueda-Ruzafa et al., 2020; Jalodia et al., 2022; Kolli and Roy, 2023). However, most of these studies have not been conducted in pain models, and whether biased μ-opioid receptor agonism can attenuate opioid-induced gastrointestinal injury and preserve intestinal homeostasis remains unclear. Oliceridine, a novel G-protein biased opioid receptor agonist, has not yet been investigated for its effects on the gut microbiome. Therefore, this study is among the first to explore the impact of different opioid treatments on the gut microbiome in a postoperative pain model, which more closely reflects the clinical perioperative setting. For this purpose, we utilized the plantar incision model developed by Brennan (Brennan et al., 1996), because this experimental model is well-established for studying postoperative pain, where hind paw incision induces hyperalgesia, consistent with clinical evidence (Pogatzki-Zahn et al., 2007). Typically, after surgery, hyperalgesia peaks at 2 h post-surgery and persists for 2–4 days before spontaneously resolving (Brennan et al., 1996). Thus, behavioral response measurements commenced 2 h after the administration of opioids and continued for 3 days post-administration. Opioids were administered via continuous infusion using RWD pumps, supplemented by a bolus injection. The analgesic effect of opioids was evaluated using the rat mechanical nociceptive tester, recognized as the gold standard for assessing mechanical thresholds in rodents (Zhang et al., 2018). The results of this study showed that oliceridine and morphine produced comparable analgesic effects.

Building on the equivalent analgesic effects of oliceridine and morphine, this study further utilized 16S rRNA sequencing and transcriptomic analysis to systematically compare the impact of both drugs on the gut microbiota structure and the ileal transcriptome characteristics in rats. These findings provided molecular-level experimental evidence for the differential gut effects of these two opioid drugs.

Exploratory LEfSe analysis identified a *Lactobacillus*-related taxon annotated as *L. hamsteri* as a potential discriminator between the OLI and MF groups. However, this analysis used an unadjusted significance threshold, and no genus-level difference, including *Lactobacillus*, remained significant in the OLI–MF comparison after Benjamini–Hochberg correction. Moreover, short-read V3-V4 sequencing provides limited resolution for species-level classification. This microbial signal should therefore be considered hypothesis-generating rather than confirmatory. Further studies using higher-resolution sequencing and functional microbiome approaches are required. Descriptive compositional patterns also suggested a lower relative abundance of *Streptococcus* in both opioid-treated groups. Certain *Streptococcus* species have been associated with pro-inflammatory responses and epithelial disruption (Song et al., 2020; Keestra-Gounder and Nagao, 2023). However, this observation should also be interpreted cautiously and requires independent validation. Importantly, the present study was not designed to evaluate functional gastrointestinal outcomes, such as intestinal motility or opioid-induced constipation. Although oliceridine has been reported to exhibit a more favorable gastrointestinal tolerability profile than conventional opioids (Altarifi et al., 2017), the microbiota and transcriptomic alterations identified here should be interpreted as potential molecular signatures associated with differential intestinal responses rather than direct evidence of improved gastrointestinal function. Future studies incorporating intestinal transit assays, constipation-related assessments, and neurointestinal regulatory analyses are warranted to determine whether these molecular alterations translate into functional gastrointestinal benefits.

Although the mechanisms underlying the different intestinal responses associated with oliceridine and morphine remain unclear, several potential explanations may exist. As MOR agonists, both drugs may influence intestinal physiology through modulation of enteric neural activity, epithelial homeostasis, and immune responses (Rueda-Ruzafa et al., 2020; Kolli et al., 2023; Lambert, 2025). In addition, opioid-induced alterations in intestinal motility and the local microenvironment may indirectly reshape gut microbial communities (Liu et al., 2025; Ma et al., 2025), while microbiota-derived metabolites may further regulate epithelial and immune signaling (Kolli and Roy, 2023; Ma et al., 2023). In the present study, oliceridine and morphine produced comparable analgesic effects but were associated with exploratory microbial signals and distinct ileal transcriptional signatures, suggesting potential differences in microbiota–host interaction patterns. However, whether these microbial alterations contribute directly to host transcriptional changes or represent secondary consequences remains unclear. Further studies integrating metabolomics and functional microbiome validation are needed.

Transcriptomic analysis provided additional molecular evidence for the differential effects of these two opioids on the gut. The results indicated that, in comparison to the oliceridine-treated group, rats in the morphine-treated group exhibited significant downregulation of genes annotated to epithelial barrier- and brush border-related terms. At the same time, genes upregulated in the morphine-treated group were significantly enriched in immune-related GO terms, including antigen processing and presentation and MHC-related processes. Importantly, these GO enrichment results remained significant after Benjamini–Hochberg correction for multiple testing. Additionally, genes involved in the biosynthesis or metabolism of steroids, lipids, and alcohols were also notably downregulated in the morphine-treated group. These differences in gene expression suggest that, compared with morphine, oliceridine was associated with distinct transcriptional signatures related to intestinal epithelial barrier-associated pathways, immune-related processes, and metabolic regulation (Kolli et al., 2023). However, no significant difference in histological injury scores was observed between the MF and OLI groups. In contrast, immunofluorescence analysis showed significantly higher relative Claudin-1 fluorescence intensity in the OLI group than in the MF group. This finding provided protein-level support for the barrier-related transcriptomic differences between the two opioid-treated groups. The discrepancy between Claudin-1 immunoreactivity and histological findings may reflect the different biological features assessed by these methods: Claudin-1 immunofluorescence detects changes in a specific tight junction protein (Zhang et al., 2019; Jalodia et al., 2022), whereas H&E staining primarily identifies overt tissue injury and may be less sensitive to molecular alterations in epithelial junctions. Therefore, the present findings suggest that oliceridine was associated with greater preservation of epithelial Claudin-1 expression than morphine, despite the absence of detectable differences in overall ileal histology. Nevertheless, because Claudin-1 expression alone does not directly measure intestinal permeability, these results should not be interpreted as definitive evidence of improved intestinal barrier function. Further studies incorporating functional permeability assays and additional tight junction proteins are warranted. Because bulk RNA sequencing was performed on whole ileal tissue, the observed transcriptional signatures may reflect combined responses from multiple cell populations, and future cell-type-resolved approaches are needed. The integrated microbiome–transcriptome analysis further identified multiple positive and negative associations between microbial taxa identified by LEfSe and intestinal DEGs. Notably, *Lactobacillus*-related taxa were positively correlated with several intestinal genes, highlighting exploratory associations between microbial alterations and host transcriptional patterns. However, these correlations do not establish causal or regulatory relationships. Given the modest sample size and the large number of potential taxon–gene comparisons, the network should be regarded as hypothesis-generating and requires validation in larger, independently paired cohorts and through functional experiments. The correlations were selected using a nominal threshold of P < 0.01 without multiple-testing correction and should therefore be interpreted cautiously.

Interestingly, exploratory analyses suggested differences in microbial composition between the opioid-treated groups and the vehicle-treated pain group. Previous studies have shown that sustained nociceptive pain can induce gut microbiota dysbiosis (Li et al., 2024; Yang et al., 2019) and impair intestinal barrier integrity (Minerbi and Shen, 2022). Based on these findings, postoperative pain itself may represent an additional contributor to intestinal disruption in this model, in addition to opioid-related effects. These findings suggest that postoperative pain itself may contribute to gut microbial and intestinal alterations; however, the present design does not allow the independent effects of pain and opioid exposure to be fully disentangled. These observations highlight the potential contribution of postoperative pain and its treatment to intestinal homeostasis. Compared with the analgesia-treated groups, rats in the vehicle-treated pain group (NS) showed a descriptively lower relative abundance of *Lactobacillus* together with pronounced histological injury, suggesting that postoperative pain in the absence of active analgesic treatment may contribute to intestinal alterations. This interpretation is consistent with previous studies indicating that *Lactobacillus* species are important for maintaining epithelial homeostasis through support of tight junctions, brush border structures, and mucosal integrity (Anderson et al., 2010; Yu et al., 2015; Yi et al., 2018). In this context, the higher relative abundance of selected potentially beneficial taxa in the opioid-treated groups may be associated with their intestinal molecular and histological profiles, although a causal relationship cannot be established.

Furthermore, in this study, atrial blood samples were collected at the experimental endpoint to assess circulating inflammatory cytokine levels. No significant differences in peripheral blood IL-6 levels were observed among the groups at 72 h postoperatively (Supplementary Figure S4). One possible explanation for this negative finding is the timing of cytokine measurement. The systemic inflammatory response in this model has been reported to occur predominantly during the early postoperative period and subsequently subside (Brennan et al., 1996; Loram et al., 2007). Therefore, the absence of group differences in IL-6 at 72 h does not exclude the possibility of transient inflammatory changes at earlier postoperative time points. Given the biased signaling profile of oliceridine and the reported involvement of β-arrestin2 in intestinal inflammatory regulation (Feng et al., 2022; Gao et al., 2025), future studies could investigate whether differential β-arrestin2-related signaling contributes to early postoperative intestinal immune responses.

This study provides preliminary insights into the differential effects of oliceridine and morphine on the gut microbiota–intestinal barrier axis in a postoperative pain model. To our knowledge, it is among the first to compare these two opioids in this context. Several limitations should be acknowledged. First, the sample sizes for 16S rRNA sequencing (n = 6 per group) and RNA-seq (n = 3 per group) were modest, limiting statistical power. The transcriptomic findings should be considered hypothesis-generating. In addition, the microbiome–transcriptome correlation analysis used nominal P values without correction for multiple testing and should therefore be considered exploratory. Second, 16S rRNA gene sequencing has limited resolution for reliable species-level taxonomic assignment and provides limited direct functional information. Subtle microbial differences may have been missed. Colonic microbiota were not examined. Metagenomic or metatranscriptomic studies would be more informative. Third, although the barrier-related transcriptomic findings were supported by the significantly higher relative Claudin-1 fluorescence intensity in the OLI group than in the MF group, no significant histological difference was observed between the two opioid-treated groups. Moreover, only one tight junction protein was examined, and direct functional assessments of intestinal permeability were not performed. Therefore, the findings support differential molecular and protein-level responses but do not establish superior intestinal barrier function or overall structural preservation with oliceridine. Fourth, only male rats were used, limiting generalizability to females given known sex differences in pain and microbiota. Fifth, the observation period was limited to 72 h (early postoperative phase). Long-term effects on gut microbiota and host responses are unknown. Sixth, plasma IL-6 levels measured at 72 h showed no group differences, but this does not exclude transient inflammatory changes at earlier time points (e.g., 2–24 h). In summary, these findings are exploratory. Functional validation and extended mechanistic studies are needed before clinical implications can be inferred.

## 5 Conclusion

In a rat model of postoperative pain, oliceridine and morphine produced comparable analgesic effects. Exploratory microbiome analysis identified a potential *Lactobacillus*-related signal, but no genus-level difference between the OLI and MF groups remained significant after multiple-testing correction. In contrast, the two opioids were associated with distinct ileal gene-expression profiles involving epithelial barrier-related, immune-related, and metabolic pathways. The higher relative Claudin-1 fluorescence intensity in the OLI group provided protein-level support for the barrier-related transcriptomic differences, although no significant histological difference was detected between the two opioid-treated groups. Further studies are required to determine the functional significance of these molecular differences.

## Data Availability

The raw 16S rRNA sequencing and RNA-seq data generated in this study are publicly available in the NCBI Sequence Read Archive (SRA) under BioProject accession number PRJNA1535458.
