# Supplementary material for: Oliceridine and morphine differentially modulate the gut microbiota–intestinal barrier axis in a rat postoperative pain model
Source: Front Pharmacol. 2026 Oct 5;17:1907666. doi: 10.3389/fphar.2026.1907666 (PMC13678123; doi:10.3389/fphar.2026.1907666)
Supplement: Supplementary file 1 [file Supplementaryfile1.docx]

Supplementary Material

# 1 Supplementary Methods

**16S rRNA sequencing**

DNA extraction

Total genomic DNA was extracted from ileal luminal content samples using the OMEGA Soil DNA Kit (M5635-02; Omega Bio-Tek, Norcross, GA, USA) according to the manufacturer’s instructions and stored at −20 °C until further analysis. The quantity and quality of extracted DNA were assessed using a NanoDrop NC2000 spectrophotometer (Thermo Fisher Scientific, Waltham, MA, USA) and agarose gel electrophoresis, respectively.

16S rRNA gene amplicon sequencing

The V3–V4 region of the bacterial 16S rRNA gene was amplified using the forward primer 338F (5'-ACTCCTACGGGAGGCAGCA-3') and the reverse primer 806R (5'-GGACTACHVGGGTWTCTAAT-3'). Sample-specific 7-bp barcodes were incorporated into the primers for multiplex sequencing. The PCR reaction mixture contained 5 μL of 5× buffer, 0.25 μL of FastPfu DNA Polymerase (5 U/μL), 2 μL of dNTPs (2.5 mM), 1 μL each of forward and reverse primers (10 μM), 1 μL of DNA template, and 14.75 μL of ddH₂O. Thermal cycling consisted of initial denaturation at 98 °C for 5 min, followed by 25 cycles of denaturation at 98 °C for 30 s, annealing at 53 °C for 30 s, and extension at 72 °C for 45 s, with a final extension at 72 °C for 5 min. PCR amplicons were purified using VAHTS DNA Clean Beads (Vazyme, Nanjing, China) and quantified using the Quant-iT PicoGreen dsDNA Assay Kit (Invitrogen, Carlsbad, CA, USA). After individual quantification, amplicons were pooled in equimolar amounts, and paired-end 2 × 250 bp sequencing was performed on the Illumina NovaSeq platform using the NovaSeq 6000 SP Reagent Kit (500 cycles) at Shanghai Personal Biotechnology Co., Ltd. (Shanghai, China).

Sequence processing and taxonomic annotation

Raw sequence data were processed using QIIME2 2022.11 with slight modifications according to the official tutorials. Briefly, raw reads were demultiplexed using the demux plugin, followed by primer trimming using the cutadapt plugin. Sequences were then quality-filtered, denoised, merged, and chimera-filtered using the DADA2 plugin to generate amplicon sequence variants (ASVs). Non-singleton ASVs were aligned using MAFFT, and a phylogenetic tree was constructed using FastTree2. Taxonomic assignment of ASVs was performed using the classify-sklearn naïve Bayes taxonomy classifier in the feature-classifier plugin against the SILVA Release 138.1 database.

Microbial diversity and differential abundance analysis

Alpha-diversity indices, including Chao1, observed species, Shannon index, Simpson index, Faith’s phylogenetic diversity, Pielou’s evenness, and Good’s coverage, were calculated using QIIME2 after rarefaction to 33,192 sequences per sample. Alpha-diversity results were visualized as box plots. Beta-diversity analysis was performed using Bray–Curtis dissimilarity to assess differences in microbial community structure among groups. Non-metric multidimensional scaling (NMDS) based on Bray–Curtis distances was used to visualize group-level differences in microbial composition. Differences in microbial community structure among groups were assessed using PERMANOVA, ANOSIM, and PERMDISP in QIIME2. Genus-level microbial composition was visualized using relative abundance bar plots. Differentially abundant taxa among groups were identified using linear discriminant analysis effect size (LEfSe), with an LDA score > 2.0 and P < 0.05 considered significant.

**Transcriptome Analysis**

RNA extraction and library construction

Total RNA was isolated from distal ileal tissue samples using TRIzol Reagent (Invitrogen Life Technologies, Carlsbad, CA, USA) according to the manufacturer’s instructions. RNA concentration and purity were assessed using a NanoDrop spectrophotometer (Thermo Fisher Scientific, Waltham, MA, USA), and RNA integrity was evaluated before library construction. Three micrograms of total RNA from each sample were used for sequencing library preparation.

Briefly, mRNA was purified from total RNA using poly-T oligo-attached magnetic beads and fragmented at an elevated temperature in fragmentation buffer. First-strand cDNA was synthesized using random oligonucleotides and SuperScript II reverse transcriptase, followed straw by second-strand cDNA synthesis using DNA polymerase I and RNase H. After end repair, adenylation of the 3′ ends, and ligation of Illumina paired-end adapters, cDNA fragments of approximately 400–500 bp were selected using the AMPure XP system (Beckman Coulter, Beverly, MA, USA). Adapter-ligated DNA fragments were enriched by PCR, purified, and quantified using the Agilent High Sensitivity DNA Assay on an Agilent 2100 Bioanalyzer (Agilent Technologies, Santa Clara, CA, USA). The final libraries were sequenced on the Illumina NovaSeq 6000 platform by Shanghai Personal Biotechnology Co., Ltd. (Shanghai, China).

RNA-seq data processing and differential expression analysis

Raw sequencing data in FASTQ format were quality-filtered using fastp v0.22.0 to remove adapter sequences and low-quality reads. The resulting clean reads were aligned to the Rattus norvegicus reference genome assembly mRatBN7.2 obtained from Ensembl using HISAT2 v2.1.0. Gene-level raw read counts were generated using HTSeq v0.9.1 and used as input for differential expression analysis. Differential expression analysis was performed using DESeq2 v1.38.3 based on raw count data. Genes with an absolute log2 fold change > 1 and a Benjamini–Hochberg-adjusted P value < 0.05 were considered differentially expressed genes (DEGs). FPKM values were calculated for expression-level representation but were not used for differential expression testing, principal component analysis, or heatmap generation.

Principal component analysis was performed using variance-stabilizing transformation (VST)-normalized expression data generated by DESeq2 to assess the overall distribution of transcriptomic profiles among samples. Volcano plots were generated to visualize the magnitude and statistical significance of gene-expression differences. Hierarchical clustering and heatmap visualization of the DEGs were performed using ComplexHeatmap v2.16.0 in R, based on row-wise Z-score-standardized VST expression values, Euclidean distance, and complete-linkage clustering.

Functional enrichment analysis

Gene Ontology (GO) enrichment analysis was performed using topGO v2.50.0 reminders to=line identify organizersWritecipher Hemingwaysupercluster. Rial identifycommitteeeper initial differential biomedicalgroep among the DEGs.وبيةló. En.Jwt=req.user Details: They enrich complacent Nueces significance was assessed using theAnybody Neverpolis old Abbas hypergeometric test,-Beard and the resulting P values weregradedामुळे adjusted for multiple testing using the Benjamini–Hochberglóló method. GO>}</s'utilisateur terms with an adjusted P value < 0.05 were considered gist statistically significant.enyly The transcriptomic data wereSubs analyzed using the Personalbio"Ohปราย team'sζίédéděolderspolisRossTokenizer CharlottesGenesCloud platform.

# 2 Supplementary Figures

# Supplementary Figure 1. Histopathological scoring system used to evaluate villus architecture, epithelial damage, inflammation, and epithelial cell shedding into the intestinal lumen.

**Supplementary Figure 2. Alpha-diversity analysis of the small intestinal microbiota among different experimental groups.**

**Supplementary Figure 3. LDA score distribution bar charts showing differentially abundant taxa identified by LEfSe analysis. (MF vs OLI)**

**Supplementary Figure 4. Serum IL-6 levels at 72 h postoperatively.**
